# Flexural Performance and Toughness Characteristics of Geogrid-Reinforced Pervious Concrete with Different Aggregate Sizes

**DOI:** 10.3390/ma14092295

**Published:** 2021-04-29

**Authors:** Xiaoyu Meng, Qinghui Jiang, Ruyan Liu

**Affiliations:** School of Civil Engineering, Wuhan University, Wuhan 430072, China; xymeng1994@whu.edu.cn (X.M.); 18202752427@163.com (R.L.)

**Keywords:** geogrid, reinforced pervious concrete, aggregate size, toughness index

## Abstract

Pervious concrete is an environmentally friendly paving material to reduce surface runoff in urban construction. However, due to low flexural strength and cracking susceptibility caused by the high porosity, pervious concrete is only used in low-volume traffic roadways and parking lots for current service. This study investigated the permeability, strength, and flexural performance of pervious concrete with different coarse aggregate size, geogrid position, and geogrid layer number. Test results indicate that the geogrid placed at an appropriate position in pervious concrete improved the permeability and compressive strength. Four-point bending tests were conducted in the laboratory to evaluate the flexural performance and toughness characteristics of pervious concrete beam. Meanwhile, this study also proposed a new evaluation method to distinguish the contribution of geogrids and concrete mixture to the flexural toughness of pervious concrete beam at the pre-peak and post-peak stages by two toughness indices. Test results indicate that geogrids improved the flexural strength, deformability, and energy absorption capability of pervious concrete beam. The geogrids placed at both one-third and two-thirds of the heights of pervious concrete beam resulted in the optimum flexural performance. Besides, the small size (5–10 mm) aggregates were conducive to providing high flexural strength for the geogrid-reinforced pervious concrete beam, while the large size (10–15 mm) aggregates played a significant role in obtaining noteworthy post-cracking performance.

## 1. Introduction

Pervious concrete has been used in urban construction due to its environmental and stormwater management benefits. Pervious concrete can allow rainfall to pass down through the ground to reduce urban flood hazards [1]. At the same time, the high permeability and aeration of pervious concrete have the potential to minimize heat island effects and reduce urban noise [2,3]. Normal pervious concrete has a compressive strength between 10 MPa and 40 MPa [4], which can meet the requirements of a pavement material. Nevertheless, limited by the defects of low flexural strength and easy cracking, pervious concrete is only used in low-volume traffic roadways and parking lots for current service.

Fiber is the main reinforcing material used to improve the flexural behavior of pervious concrete. Considerable efforts have been devoted to investigating the influence of fiber types [5,6], sizes [7], volume fractions [8], and treatments [9] on the mechanical properties of the fiber-reinforced pervious concrete. Fibers can reduce the brittleness, and improve the flexural strength, deformation capacity, and energy dissipation of pervious concrete. However, placing fibers in pervious concrete still faces challenges due to the insufficient fiber-matrix bonding interface caused by high porosity. The flexural behavior of pervious concrete needs to be further improved.

Geogrid has been playing an important role in solving geotechnical problems due to its high strength and elongation ratio [10,11,12,13,14]. In pavement applications, geogrid is commonly used to provide safety for traffic and increase the service life of roads. Sun et al. [15] showed that a geogrid placed at unpaved roads reduced both the base course and subgrade permanent deformations and changed the radial stress distributions within the base course and subgrade. Geogrid has been also employed as an anti-reflective-cracking system in the reconstruction of pavement. Khodaii et al. [16] studied the effects of geosynthetic reinforcement on mitigating reflection cracking in asphalt overlays and found that a geogrid placed at one-third the height of overlay thickness could obtain the optimum benefit. Hadi and Al-Hedad [17] investigated the flexural fatigue behavior of geogrid-reinforced concrete pavements subjected to cyclic loads and developed an equation for designing geogrid-reinforced concrete pavements. Meski and Chehab [18] conducted four-point bending tests of geogrid-reinforced concrete beam and assessed the feasibility of placing geogrids in thin concrete overlays. Tang et al. [19] showed a failure model of geogrid-reinforced Portland cement concrete. Al-Hedad and Hadi [20] showed that geogrid-reinforced concrete slab had improved flexural performance and cracking resistance. Mohamed et al. [21] conducted a four-point bending test on geogrid-reinforced concrete slabs and indicated that triaxial geogrids could provide lower deflection values and higher first-crack load values for geogrid-reinforced concrete slabs. Meng et al. [22] studied the flexural behavior of geogrid-reinforced pervious concrete beam with a different geogrid layer number at various positions. The studies of geogrid-reinforced pavement mainly focus on asphalt or concrete pavement at present. Little research has been done on geogrid-reinforced pervious concrete pavement. On the one hand, geogrid can provide additional tensile strength for pervious concrete to restrain cracking. On the other hand, water can pass though the aperture structure of geogrid without affecting the permeability of pervious concrete. Moreover, as a polymeric material, geogrid has a good corrosion resistance, and would not be rusted in water like steel. Obviously, these benefits make it feasible to use geogrid as reinforcement in pervious concrete.

Flexural toughness is a major index to reflect the flexural behavior of geogrid-reinforced pervious concrete. However, the accepted flexural toughness evaluation methods are based on fiber-reinforced concrete [23,24,25]. Besides energy absorption, dimensionless indices related to energy absorption capacity, and equivalent flexural strengths as prescribed by post-cracking deflections or other parameters are also usually used to describe the flexural behavior of fiber-reinforced concrete. ASTM C1018 [26], JSCE SF-4 [27], and post-crack strength (PCS) [28] are common methods to evaluate the flexural toughness of fiber-reinforced pervious concrete by bending test with unnotched beam. The dimensionless indices in the ASTM C1018 method [26] can effectively evaluate the flexural toughness of specimens at different stages without the limit of the loading conditions and specimen sizes. Nevertheless, there are some difficulties in determining the initial crack point. The JSCE SF-4 method [27] used the equivalent flexural strength up to a certain vertical displacement to evaluate the flexural toughness of beams. This method avoided the influence of first crack vertical displacement on toughness evaluation. However, using the entire area under the load-vertical displacement curve made it difficult to distinguish the contributions of concrete and reinforcement to the flexural toughness. Meanwhile, the dimensional index resulted in specimens of different sizes or under different loading conditions being incomparable to each other. Benthia and Trottier [28] proposed the PCS method to evaluate flexural toughness by calculating post-peak equivalent flexural strength. Besides the benefit that the first crack vertical displacement was not considered, the PCS method highlighted the effect of reinforcement on post-peak performance. However, the effect of the reinforcement on the pre-peak stage was not considered. Although the flexural toughness evaluation methods of fiber-reinforced concrete can be used as references for geogrid-reinforced pervious concrete, clearly, an alternative method is also needed for geogrid-reinforced pervious concrete.

So far, studies on the use of geogrid as reinforcement in pervious concrete to improve flexural performance are rare. Therefore, this study investigated the effect of aggregate size, geogrid position, and geogrid layer number on the flexural behavior of pervious concrete. In order to describe the flexural toughness characteristics of geogrid-reinforced pervious concrete, a new evaluation method was preliminarily introduced and used to evaluate the four-point bending test results. The outstanding advantage of this method is that it can distinguish the respective contributions of the geogrids and the concrete mixture to the flexural toughness of pervious concrete at the pre-peak and post-peak stages during the bending tests.

## 2. Materials and Methods

### 2.1. Mixture Proportions and Materials

**Figure 1:**
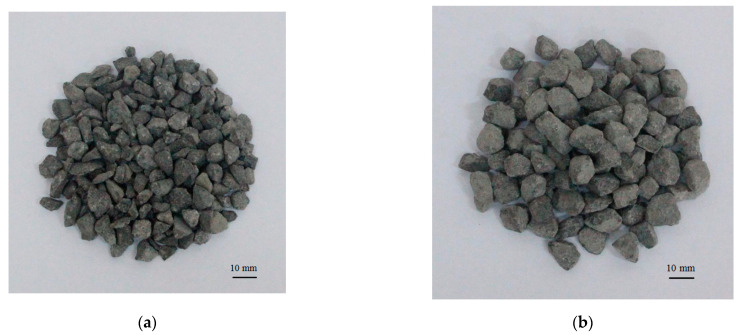
Photos of the two types of coarse aggregates: (**a**) 5–10 mm, (**b**) 10–15 mm.

The mix proportion of pervious concrete with single gradation coarse aggregates was calculated by volume method, as shown in Table 1. Type I Portland cement was used as the main cement-based material, with a density of 3100 kg/m^3^. Fly ash with specific surface area of 650 m^2^/kg was used as the active mineral to improve the strength of pervious concrete. There were no fine aggregates in the pervious concrete cast in this study. Two types of coarse aggregates were used to investigate the effect of coarse aggregates’ size on the flexural behavior of the geogrid-reinforced pervious concrete. Figure 1 shows the small size (5–10 mm) coarse aggregates and large size (10–15 mm) coarse aggregates.

**Figure 2:**
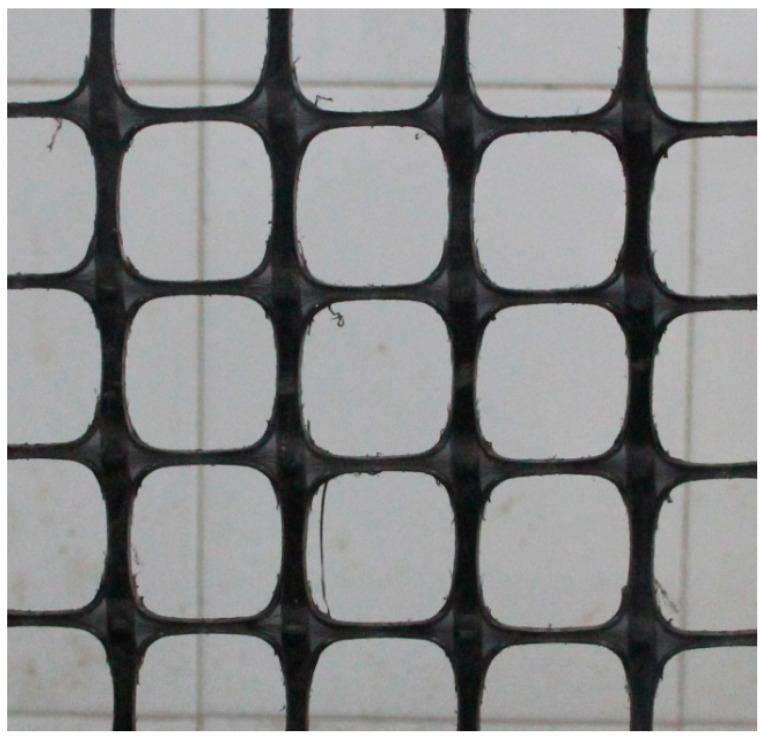
Biaxial geogrid.

**Figure 3:**
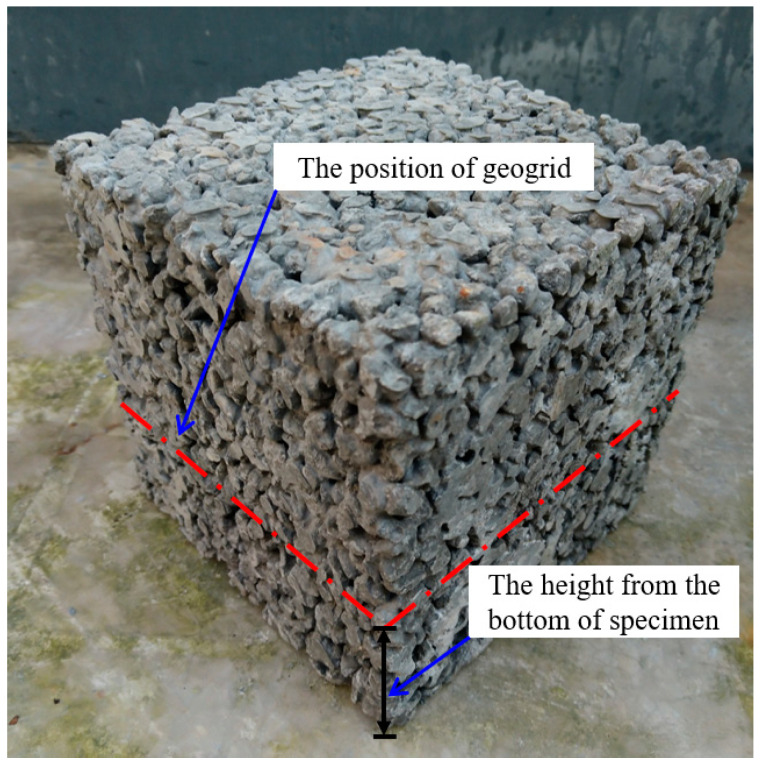
The position of geogrid in specimen.

Polypropylene biaxial geogrid with 40 mm × 40 mm apertures and a tensile strength of 21 kN/m was used as reinforcement in pervious concrete as shown in Figure 2. Therefore, the ratios of the geogrid aperture to the maximum diameter of small-size aggregate and large-size aggregate were 4.0 and 2.7, respectively. Table 2 shows the physical and mechanical properties of the geogrid obtained by laboratory tests. In order to examine the effects of coarse aggregate size, geogrid position, and geogrid layer number on the permeability, compressive strength, and flexural behavior of pervious concrete, twelve types of specimens in different tests were determined as shown in Table 3. The test results were averaged from three replicates of each specimen type. The geogrid position means the height from the bottom of the specimen, as shown in Figure 3.

### 2.2. Sample Preparation

The satisfactory use of geogrid-reinforced pervious concrete as a composite structure depends mainly on the good interlock and bond between geogrids and concrete. To cast the geogrid-reinforced samples, a lower concrete layer was first poured to the marked location in the mold and compacted with a steel rod. Then, the flat geogrid layer was carefully placed at the marked position. Finally, an upper layer of concrete was poured above the geogrid with compaction. Most importantly, the aperture of the geogrid should be filled with aggregates as much as possible during the pouring process to ensure the proper bonding between the lower and upper layers of concrete. The cast specimens were maintained under a laboratory condition for 24 h. Then, the specimens were demolded and placed in a standard curing room for 28 days, with continuous wetting cycles at a temperature of 23 ± 2 °C and relative humidity of 95%.

Similar to common pervious concrete pavement, the concrete formwork should be prepared first when building geogrid-reinforced pervious concrete pavement in the field. Then, the position of the geogrid is marked on the formwork. In the process of building the geogrid-reinforced pervious concrete pavement, the lower pervious concrete layer is first poured to the marked location with compaction. Thereafter, the geogrid is installed above the lower pervious concrete layer. In order to ensure the accurate installation position of the geogrid, the edges of the geogrid are fixed at the marked position on the formwork by steel wire. Then, the upper pervious concrete layer is poured above the geogrid with compacting to the design elevation. It should be mentioned that geogrids from different rolls should be overlapped and bound to ensure integrity when the length of single-roll geogrid is limited.

### 2.3. Experimental Procedure

**Figure 4:**
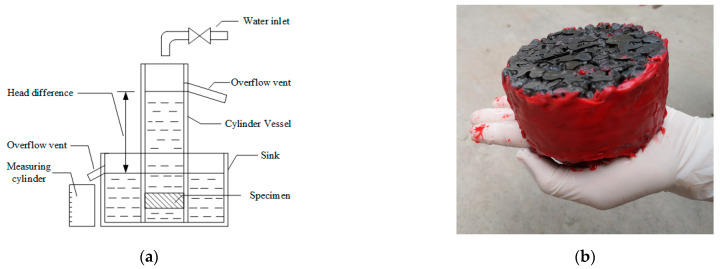
Permeability test: (**a**) test setup, (**b**) specimen.

Based on Darcy’s law, the equipment for the permeability test was designed as shown in Figure 4a. Unreinforced and geogrid-reinforced cylindrical specimens with a size of Φ 100 mm × 50 mm were used in the permeability tests. Industrial butter was smeared around the sides of specimens to ensure that the water only permeated from the upper surface to the lower surface of specimens, as shown in Figure 4b. The permeability coefficient of pervious concrete was calculated as follows:

(1) $$k=\frac{QL}{AHt}$$

where *k* is the permeability coefficient (mm/s); *Q* is the permeating water in *t* seconds (mm^3^); *L* is the thickness of specimen (mm); *A* is the cross area of the specimen (mm^2^); *H* is the water head difference (mm); and *t* is the time (s).

**Figure 5:**
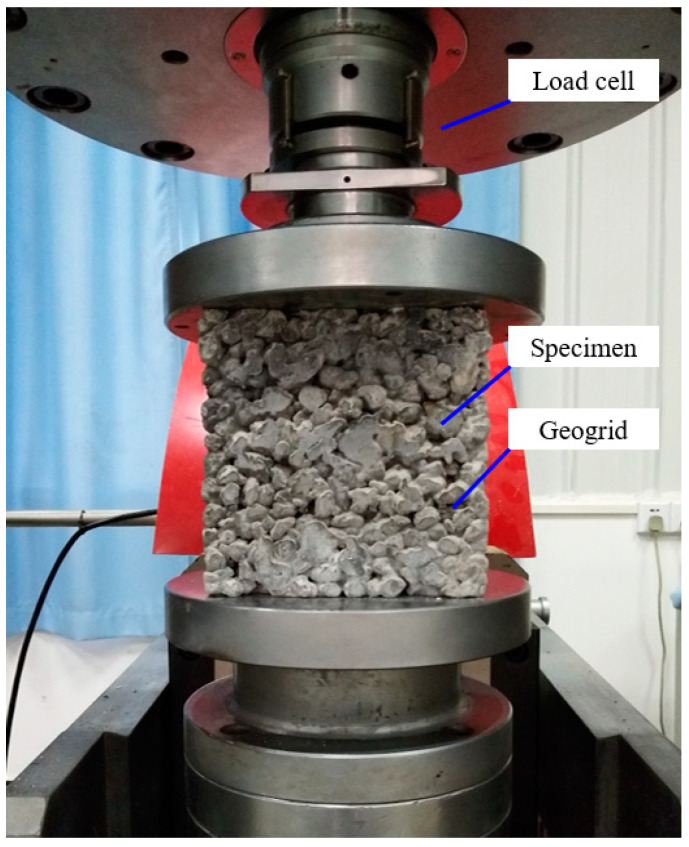
The unconfined compressive strength test setup.

Unconfined compressive strength tests were performed on the pervious concrete cubes with different coarse aggregate sizes, geogrid positions, and geogrid layer numbers at a curing age of 28 days. Figure 5 shows the unconfined compressive strength test setup. The height of the pervious concrete cube was 150 mm. During the compressive strength test, an axial loading with a constant rate of 10 kN/s acted on the specimens until failure.

**Figure 6:**
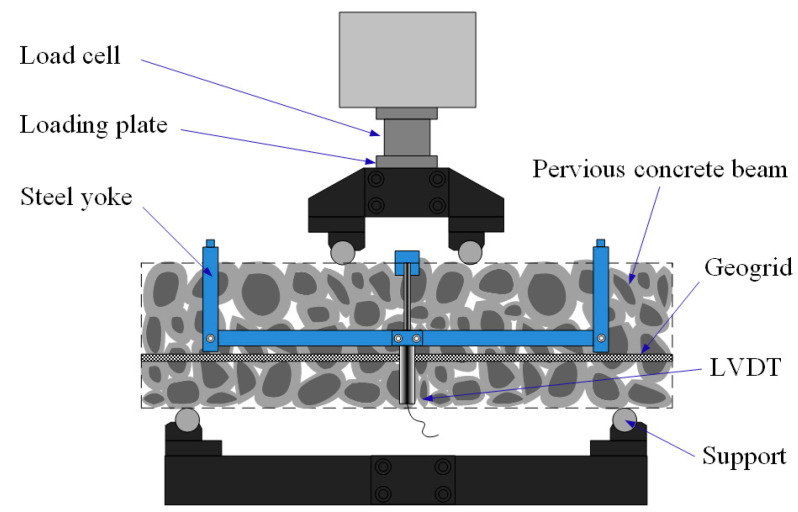
Four-point bending test setup.

The four-point bending tests were carried out using a hydraulic testing machine with a maximum load capacity of 1500 kN to evaluate the flexural behavior of the geogrid-reinforced beams. Figure 6 shows the four-point bending test setup. The dimension of the beam was 550 mm × 150 mm × 150 mm. The span length between the two lower supports was 450 mm, while the length between the two upper supports was 150 mm. It is worth noting that the casting surface of beam should not be used as a supporting surface, due to its uneven surface. The beam was loaded with a displacement rate of 0.01 mm/s. The vertical displacement at the mid-span of beam was obtained by a linear variable differential transformer (LVDT), which was installed on the steel yoke [29]. This study tested 36 beams with different coarse aggregate sizes, geogrid positions, and geogrid layer numbers, including three replicates for each type of beam.

## 3. Permeability and Compressive Strength

### 3.1. Permeability Coefficient

**Figure 7:**
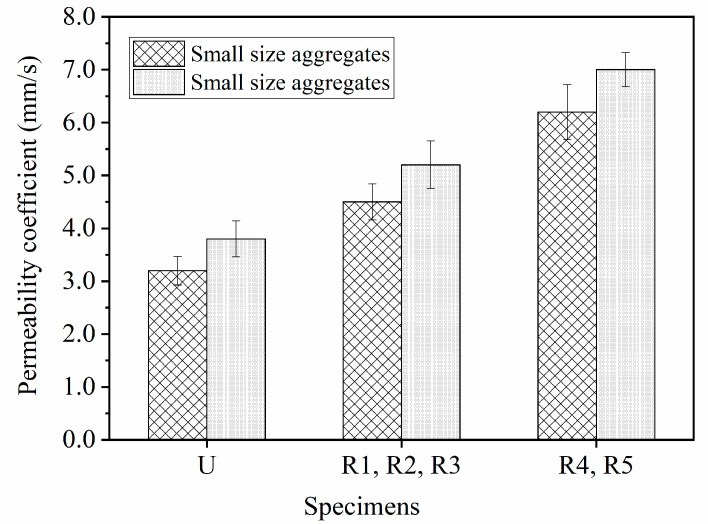
The permeability coefficients of pervious concrete specimens.

The permeability coefficient is the basic feature to reflect the permeability of pervious concrete. Figure 7 shows the permeability coefficients of pervious concrete specimens with different coarse aggregate size, geogrid position, and geogrid layer number. Test results indicate that the specimens with large-size aggregates had higher permeability coefficient than those with small-size aggregates. In pervious concrete, the coarse aggregates wrapped in cement paste bond with each other to form a porous structure. The number of conjoint points between aggregates determines the permeability of pervious concrete. As compared with the small-size aggregates, there are fewer conjoint points between the large-size aggregates in pervious concrete, which leads to the higher permeability. The permeability coefficients of unreinforced pervious concrete specimens with small- and large-size aggregates were 3.2 mm/s and 3.8 mm/s, respectively.

The placement of geogrid decreased the conjoint points between aggregates in geogrid-reinforced pervious concrete. As a result, Figure 7 shows that the permeability of pervious concrete increased when the geogrid was placed in pervious concrete. It is also observed from Figure 7 that the pervious concrete specimens reinforced with the same geogrid layer number had similar permeability. When one layer of geogrid was placed in the pervious concrete, the permeability coefficients of specimens with small- and large-size aggregates were 4.5 mm/s and 5.2 mm/s, respectively. When two layers of geogrids were placed in pervious concrete, the conjoint points of aggregates were further reduced. As a result, the permeability coefficients of specimens with small- and large-size aggregates increased to 6.2 mm/s and 7.0 mm/s, respectively. Obviously, the permeability of the pervious concrete specimens increased with the increase of the number of geogrid layers. Therefore, the placement of geogrid could improve the drainage performance of pervious concrete. Although fewer conjoint points between aggregates would bring loss of strength, the geogrid(s) could still provide favorable mechanical properties for pervious concrete, which will be discussed in the following sections.

### 3.2. Compressive Strength

**Figure 8:**
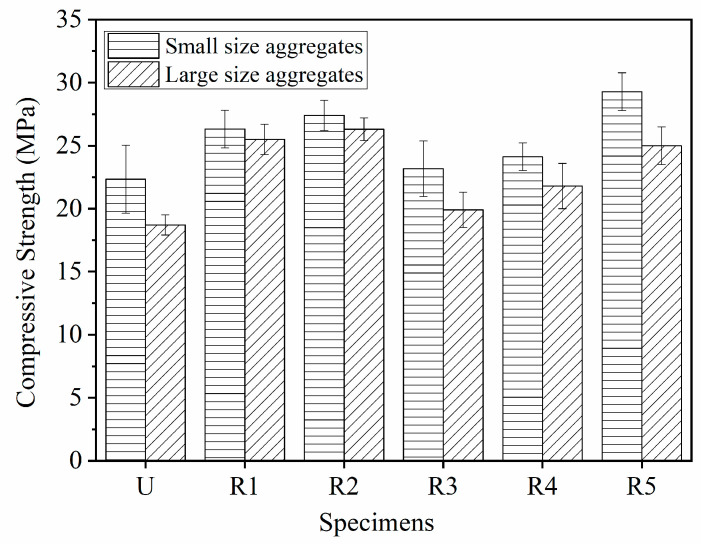
Compressive strength of pervious concrete specimens.

Figure 8 shows the compressive strength of pervious concrete specimens at a curing time of 28 days. The compressive strengths of pervious concrete with small-size aggregates and large-size aggregates were 22.3 MPa and 18.7 MPa, respectively. Apparently, the number of conjoint points between aggregates will influence the compressive strength of pervious concrete. Owing to having more conjoint points, the pervious concrete with small-size aggregates had higher compressive strength than that with large-size aggregates.

**Figure 9:**
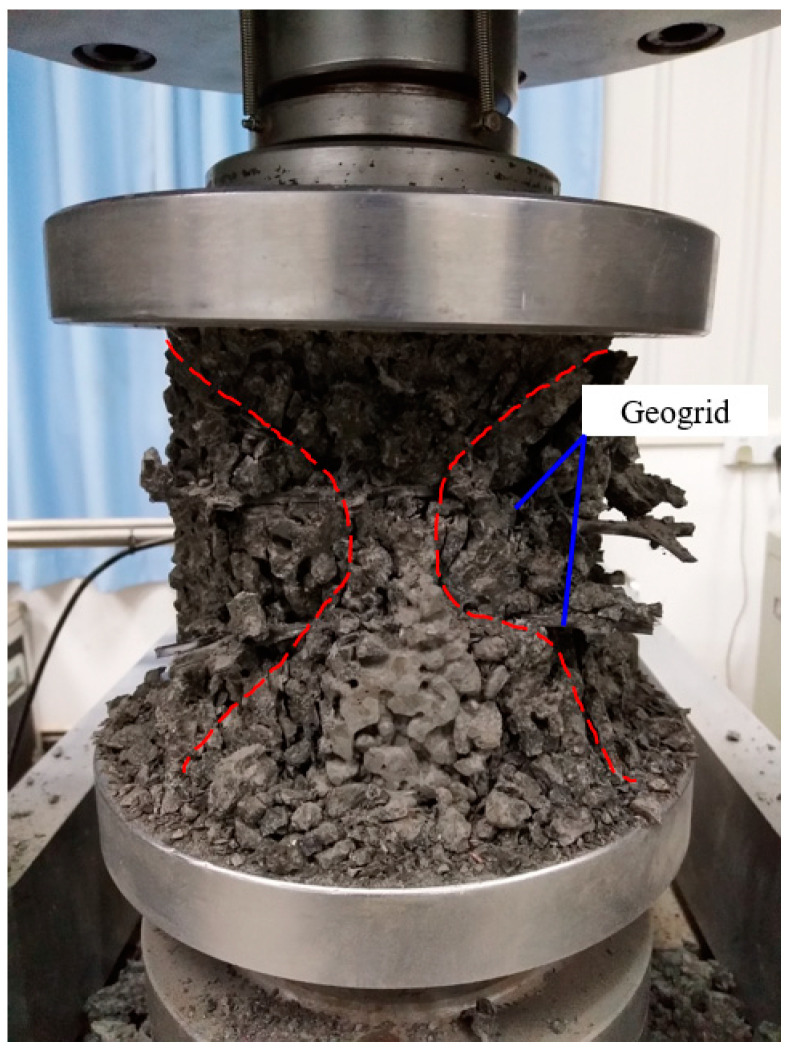
The typical failure model of a specimen under uniaxial loading.

Horizontal tensile stress occurred in pervious concrete under uniaxial loading. Then, the vertical micro cracks generated, propagated, and coalesced with other micro cracks until the specimen’s failure. When geogrid was placed in pervious concrete, the geogrid brought additional tensile strength to the pervious concrete with the help of the geogrid’s aperture structure; it also reduced the conjoint points between aggregates. While the geogrid redistributed the stress to restrain the generation and propagation of cracks, the gain of compressive strength provided by geogrids could offset the loss of strength caused by the decreased conjoint points. As a result, the geogrid-reinforced pervious concrete has improved compressive strength as compared with the unreinforced pervious concrete. Moreover, the geogrid position and layer number could influence the compressive strength of pervious concrete. Figure 8 shows that placing the geogrid at the one-third height of the pervious concrete cube obtained the highest compressive strength when one layer of geogrid was placed. As compared with the unreinforced pervious concrete specimen, the compressive strengths of reinforced specimens SR2 and LR2 increased to 27.4 MPa and 26.3 MPa, respectively. During the uniaxial loading process, the upper- and lower-end faces of pervious concrete cubes were subjected to the friction of the loading plate. The farther away from the upper- and lower-end faces, the less affected by friction was the part in the specimen. The middle part of the specimen will produce the maximum lateral displacement, which results in the failure model of pervious concrete as shown in Figure 9. As compared with specimen R4, the geogrids in R5 were closer to the bulge part on the middle of the specimens. Therefore, the specimen R5 had higher compressive strength than R4. General speaking, Figure 8 shows that the pervious concrete reinforced with one layer of geogrid had higher compressive strength than those reinforced with two layers of geogrid. This resulted mainly because the gain of compressive strength provided by the second geogrid could not offset the loss of strength caused by the decreased conjoint points. However, it should be noticed that specimen SR5 had higher compressive strength than the specimens reinforced with one layer of geogrid. The possible reasons are listed below. Firstly, as compared with the large-size aggregates, the small-size aggregates had more conjoint points, which resulted in the increased compressive strength. Secondly, the positions of geogrid in SR5 were conducive to restraining the tensile stress. The combined effect of the aggregate size, geogrid position, and layer number led to the high compressive strength of the specimen SR5.

## 4. Load-Vertical Displacement Curves

### 4.1. Effect of Geogrid Position and Layer Number

**Figure 10:**
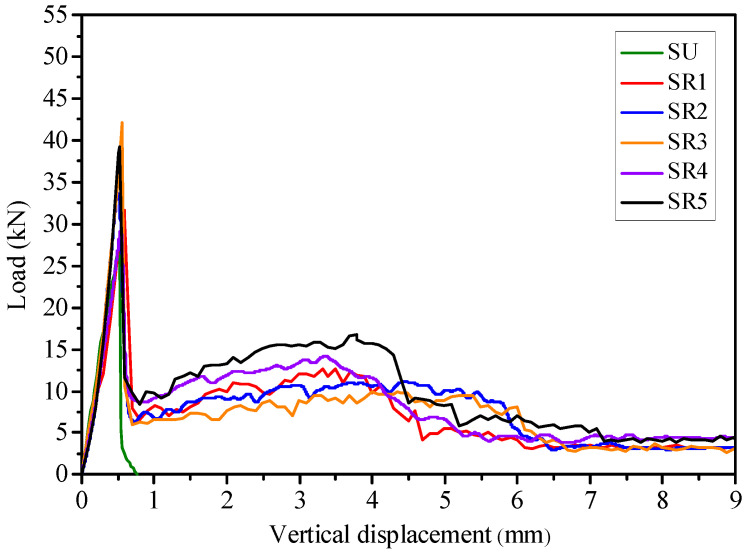
Load-vertical displacement curve of the pervious concrete beams with small-size aggregates.

**Figure 11:**
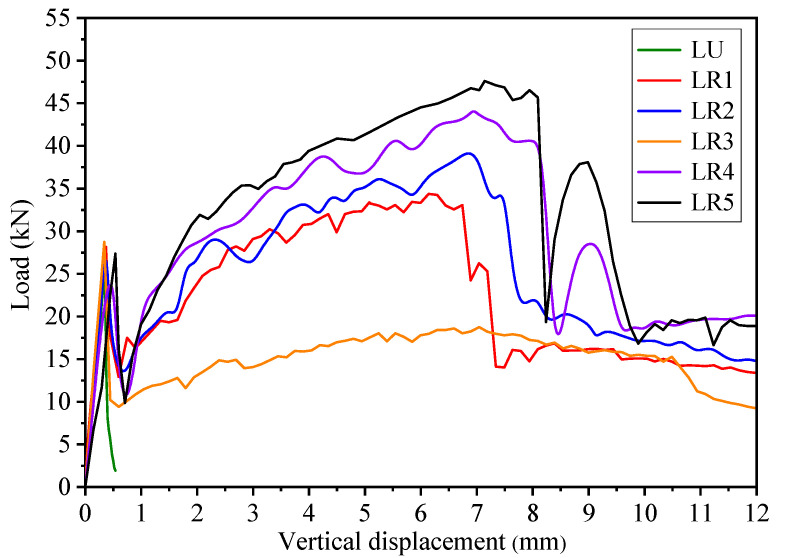
Load-vertical displacement curve of the pervious concrete beams with large-size aggregates.

The load-vertical displacement curves of the pervious concrete beams with small-size aggregates and large-size aggregates are shown in Figure 10 and Figure 11, respectively. The load-vertical displacement curves show that the unreinforced pervious concrete beam was brittle and had no post-cracking performance. The load of the unreinforced beams SU and LU increased linearly with the increase of vertical displacement, followed by a steep drop. The flexural strengths of the beams SU and LU were 3.7 MPa and 3.4 MPa, respectively.

As compared with the unreinforced beams, the flexural strength and the post-cracking performance of the geogrid-reinforced pervious concrete beams were improved significantly. For instance, Figure 10 shows that the flexural strengths of the beams SR1, SR2, and SR3 increased to 4.2 MPa, 5.1 MPa, and 5.6 MPa, respectively, when one layer of geogrid was placed. As opposed to the load of the unreinforced beam, which rapidly dropped after the first peak load, the load of the geogrid-reinforced beam began to rise when the load reduced to a certain level. A second peak load occurred on the load-vertical displacement curve of the reinforced beam due to the placement of geogrid. This remarkable post-cracking performance of the geogrid-reinforced pervious concrete beam resulted from the good interlock between geogrids and aggregates. The geogrid well interlocked with aggregates possessed a crack-bridging effect, which explained the preservation of the reinforced beam’s initial stiffness and the appearance of post-cracking performance.

Figure 10 and Figure 11 show that the curves of the pervious concrete beam reinforced with geogrid at one-third height shifted upward significantly compared to other beams reinforced with one layer of geogrid. It is obvious that the one-third height of pervious concrete beam is the optimum position for one layer of geogrid to obtain good post-cracking performance. When the geogrid was placed at a relatively high position, such as the middle height of the pervious concrete beam, the geogrid was far away from the tension zone of the beam during the bending test. The stress would be clustered in the small range of geogrid in mid-span [30]. As a result, the dissipation effect of the geogrid on stress concentration was weakened. When the geogrid was placed at a relatively low position, the lower pervious concrete layer would not interlock well with the geogrid due to the thinness. If the lower pervious concrete layer is too thin, the geogrid may be peeled off, subject to the increasing stress. Obviously, the lower pervious concrete should have a sufficient thickness to obtain good interlock between geogrid and pervious concrete. Therefore, the pervious concrete beams reinforced with geogrid at one-third height had better post-cracking performance than the beams reinforced with geogrid at one-fourth height, thanks to the good interlock between the geogrid and pervious concrete. When two layers of geogrids were placed in the pervious concrete beam, the load of the beam during the post-peak stage was higher than when one layer of geogrid was placed, as shown in Figure 10 and Figure 11. The lower layer of geogrid in pervious concrete undertook the tension stress and transferred the stress to the surrounding pervious concrete by the geogrid’s aperture structure, which redistributed the stress and improved the flexural performance. Like the pressurized bar in normal concrete, the upper layer of geogrid in pervious concrete could also improve the flexural behavior, as well as bear compression. The upper layer of geogrid provided additional toughness for the pervious concrete beam to absorb more energy during the bending test. The lower and upper layers of geogrids worked together to restrain cracking and produce a crack-bridging effect, resulted in the remarkable post-cracking performance of pervious concrete. The load-vertical displacement curves show that placing the geogrids at both the one-third and two-thirds heights of pervious concrete beam could obtain the optimum post-cracking performance.

### 4.2. Effect of Different Aggregate Size

The aggregate size is an important factor to influence the flexural behavior of pervious concrete beam. Figure 10 and Figure 11 show that the pervious concrete beams with small-size aggregates had higher flexural strength than those with large-size aggregates. Meanwhile, as compared with the unreinforced beams LU and SU, the flexural strengths of geogrid-reinforced pervious concrete beams with large- and small-size aggregates had increased by 6% to 14% and 14% to 52%, respectively. It is obvious that the small-size aggregates are more beneficial to improve the flexural strength of the geogrid-reinforced pervious concrete beam than the large-size aggregates. When small-size aggregates were used in pervious concrete, more aggregates could fill in the apertures of geogrid as compared with the large-size aggregates. The increased conjoint points between aggregates and geogrids resulted in a better interlocking effect. Therefore, the good interlocking between small-size aggregates and geogrids significantly improved the flexural strength of pervious concrete beam.

Figure 10 and Figure 11 also show that the coarse aggregate size influenced the shape of the load-vertical displacement curve of the pervious concrete beam. On the one hand, the load-vertical displacement curves of the geogrid-reinforced pervious concrete beams with large-size aggregates had an upward shift at the post-peak stage as compared with those with small-size aggregates. The second peak load of the reinforced beams with large-size aggregates was almost 3 times higher than those beams with small-size aggregates. For example, when the geogrids were placed at both the one-third and two-thirds heights of the beam, the second peak load of beam LR5 was 47.6 kN, while that of beam SR5 was only 16.7 kN. On the other hand, the second peak load of the reinforced beam with large-size aggregates was higher than the first peak load, which was contrary to the beam with small-size aggregates. Apparently, the geogrid-reinforced beam with large-size aggregates had remarkable post-cracking performance.

Different aggregate sizes had different influences on the flexural behavior of the geogrid-reinforced pervious concrete beam at pre-peak and post-peak stages during the bending tests. The small-size aggregates played an important role at the pre-peak stage of the geogrid-reinforced pervious concrete beam. The small-size aggregates were interlocked well with the geogrids in the pervious concrete beam to resist brittle failure and improve the flexural strength. While the good interlock between small-size aggregates and geogrid absorbed a lot of energy at the pre-peak stage, the energy absorption capacity of geogrid was diminished after the first peak load, which caused a lower second peak load compared to the first peak load. As mentioned earlier, the pre-peak flexural performance of beams with large-size aggregates was not as good as that with small-size aggregates. As compared with the small-size aggregates, the large-size aggregates played a smaller role in improving the flexural strength of the geogrid-reinforced beam due to the poor interlock between geogrids and large-size aggregates. Since there was little energy absorption during the elastic stage of the geogrid-reinforced beam with large-size aggregates, the geogrid provided more tensile stress to absorb energy with the generation of cracks at the post-peak stage. As a result, the post-cracking performance of the reinforced beam with large-size aggregates was particularly outstanding, with a higher second peak load than first peak load. In a nutshell, while the geogrid-reinforced pervious concrete beam with small-size aggregates obtained high flexural strength, the reinforced beam with large-size aggregates had remarkable post-cracking performance.

### 4.3. Failure Model of Geogrid

Pervious concrete is a porous structure formed by the bonding of coarse aggregates wrapped in cement paste. When geogrid was placed in pervious concrete, the aggregates filled into the apertures of the geogrid and bonded with the ribs of the geogrid to form a composite structure. Therefore, the interaction between geogrids and pervious concrete could directly affect the performance of geogrid-reinforced pervious concrete. There were friction, bonding, and interlocking interactions between the geogrid and pervious concrete. The polypropylene geogrid had a smooth surface, which led to a relatively low bonding strength and friction between the ribs of the geogrid and the pervious concrete. The geogrid being placed tightly in the porous structure of pervious concrete mainly relied on the interlocking effect between geogrid and aggregates. The use of geogrid at an appropriate position could provide additional tension stress to improve the strength and restrain the cracking of pervious concrete.

**Figure 12:**
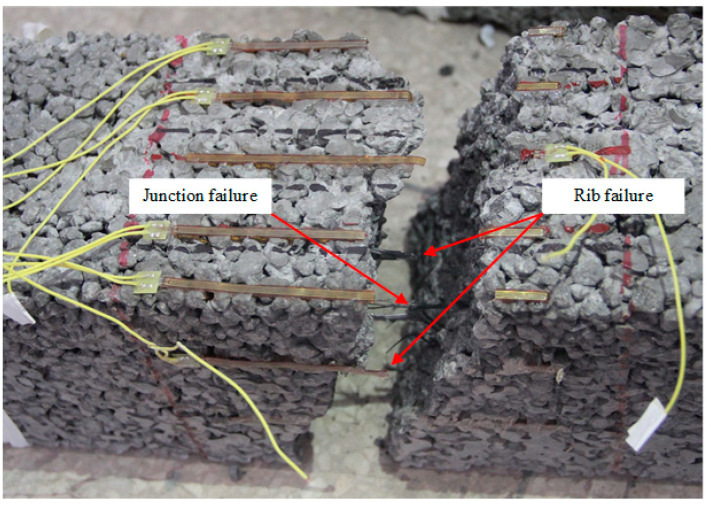
The failure model of geogrid in the bending test.

The load-vertical displacement curves show that the unreinforced pervious concrete beam was brittle and had no post-cracking performance. As compared with the unreinforced pervious concrete beams, the geogrid-reinforced pervious concrete beams had improved flexural strength and remarkable post-cracking performance. This illustrates that the geogrids worked during both the pre-peak and post-peak stages of the reinforced pervious concrete beam in bending tests. During the pre-peak stage, geogrids and pervious concrete worked together to improve the stiffness, delay the cracking, and improve the flexural strength of the reinforced beam. After cracking, the geogrids outside the crack were fixed and clamped tightly by the interlock between geogrids and concrete. At the same time, the ribs and junctions of geogrid inside the crack withstood the tension and bending moment under loading. The ribs of geogrids were stretched and deformed gradually until failure. Figure 12 clearly shows the failure model of geogrid placed in pervious concrete. On closer inspection, the ribs and junctions of geogrid on the pervious concrete fracture surface were snapped. Besides this, the geogrid in pervious concrete beam had little slippage and pullout. These provided effective evidence for the interlocking effect between geogrid and aggregates. Obviously, the interlock between geogrid and pervious concrete was the primary mechanism for the improved flexural behavior of the geogrid-reinforced pervious concrete beam. The aggregate size, geogrid position, and geogrid layer number were important factors to influence the interlocking effect between geogrid and pervious concrete.

## 5. Toughness and Toughness Index

### 5.1. Characteristics of Toughness Indices

Toughness indices have been commonly used to evaluate the flexural performance of fiber-reinforced concrete beams. Toughness is referred to as the area under the load-vertical displacement curve during the bending test, which represents the amount of energy that a specimen is able to absorb during loading up to a certain vertical displacement [31]. Moreover, the toughness index of fiber-reinforced concrete has various definitions in different evaluation methods. Similar to fiber-reinforced concrete, geogrid-reinforced concrete is also a composite structure. Geogrids are flat, placed at certain positions in geogrid-reinforced concrete, while fibers are distributed in three-dimensional space and form a net structure in fiber-reinforced concrete. While there is no evaluation method for geogrid-reinforced concrete, the flexural toughness evaluation methods of fiber-reinforced concrete can be used as references for the geogrid-reinforced pervious concrete. Owing to its simple conduct in many practical situations, the bending test is the most popular way to characterize the flexural toughness of concrete beam. Consequently, four-point bending tests were carried out in this study to evaluate the flexural toughness of the geogrid-reinforced pervious concrete beam.

**Figure 13:**
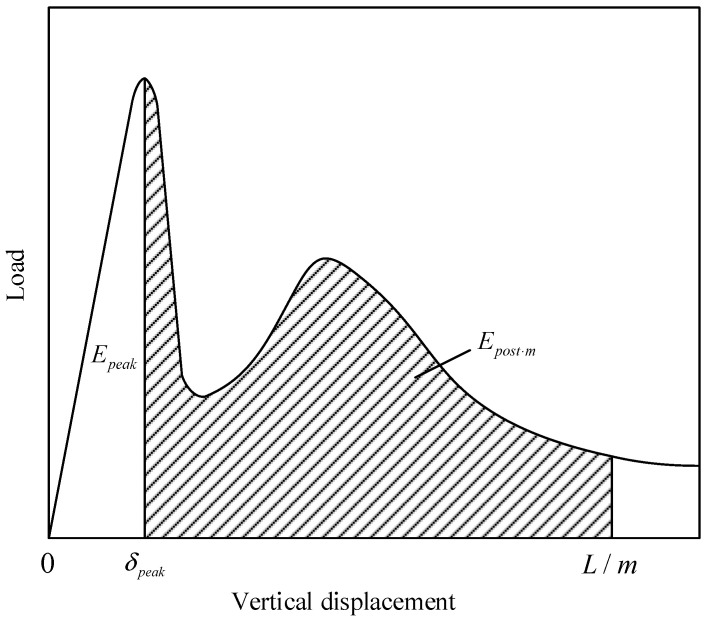
Definition of flexural toughness in this study.

Section 4 clearly indicates that the geogrid played different roles before and after the first peak load in the flexural performance of pervious concrete. Distinguishing the contribution of geogrids to flexural performance at the pre-peak and post-peak stages is of great significance for the geogrid-reinforced pervious concrete. The equivalent flexural strength was used in the JSCE SF-4 [27] and PCS [28] methods to evaluate the toughness of the concrete beams. Based on these, the equivalent flexural strength at pre-peak and post-peak stages of pervious concrete beam are calculated as follows (Figure 13):

(2) $$f_{p,r}=\frac{E_{peak}L}{\delta_{peak}bh^{2}}$$

(3) $$f_{m,r}=\frac{E_{post\cdot m}L}{(L/m-\delta_{peak})bh^{2}}$$

where $f_{p,r}$ and $f_{m,r}$ are equivalent flexural strengths at the pre-peak and post-peak stages, respectively (MPa); L is the span (mm); b is the width of the beam (mm); h is the height of the beam (mm); $\delta_{peak}$ is the vertical displacement at peak load (mm); $E_{peak}$ is the area under the load-vertical displacement curve before $\delta_{peak}$ (J); $E_{post\cdot m}$ is the area under the load-vertical displacement curve from $\delta_{peak}$ to L/m (J); and m is 50. It should be noted that the value of *m* was set to 150 in the JSCE SF-4 [27], PCS [28], and ASTM C1069 [32] methods. Figure 10 and Figure 11 show that the load of geogrid-reinforced concrete beam could not reach its second peak value at 3 mm (i.e., L = 450 mm, m = 150) in the load-vertical displacement curve. Apparently, $f_{m,r}$ could not completely reflect the post-cracking performance of the geogrid-reinforced beam. Thus, this study set *m* as 50 instead of 150 to guarantee sufficient consideration of the post-peak stage in the flexural toughness evaluation of the geogrid-reinforced beam.

The pre-peak equivalent flexural strength of plain pervious concrete ($f_{p,u}$) only depends on the property of plain concrete itself, so it is used as a baseline to evaluate the contribution of geogrid to the flexural performance of pervious concrete. The ratio of pre-peak and post-peak equivalent flexural strength of geogrid-reinforced pervious concrete beam to the pre-peak equivalent flexural strength of plain pervious concrete beam could distinguish the effect of geogrid on the flexural performance of pervious concrete at the pre-peak and post-peak stages. Therefore, the dimensionless indices $R_{e,p}$ and $R_{e,m}$ are defined as follows:

(4) $$R_{e,p}=\frac{f_{p,r}}{f_{p,u}}\times 100\%$$

(5) $$R_{e,m}=\frac{f_{m,r}}{f_{p,u}}\times 100\%$$

With the help of these two indices, the contribution of the embedded geogrid to the flexural performance of pervious concrete beam, including the energy absorption provided by the interlock between geogrid and concrete and the crack-bridging effect produced by geogrid, could be clearly characterized. $R_{e,p}$ represents the influence of the geogrid on the flexural strength and brittleness of pervious concrete beam, while $R_{e,m}$ reflects the post-cracking performance of the geogrid-reinforced pervious concrete beam. It is worthwhile to mention that the flexural toughness indices proposed in this study are not constrained by the initial crack point. The flexural performance of specimens of different sizes could be compared and evaluated thanks to the dimensionless indices.

### 5.2. Index $R_{e,p}$ of Geogrid-Reinforced Beam

**Figure 14:**
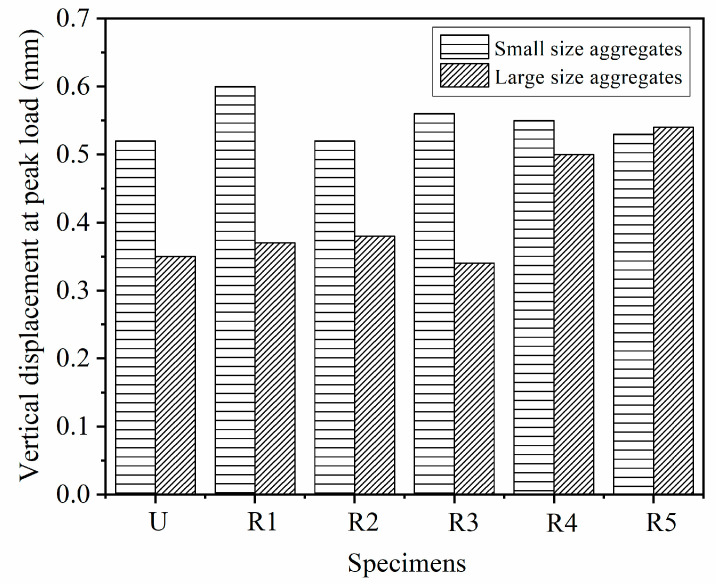
Vertical displacement at peak load of geogrid-reinforced beam.

The index $R_{e,p}$ reflects the contribution of geogrid to the flexural performance of pervious concrete beam at the pre-peak stage. Table 4 shows that the index $R_{e,p}$ of the geogrid-reinforced pervious concrete beam was higher than 1. Apparently, the placement of geogrid improved the flexural strength of pervious concrete beam. Besides, Figure 14 shows that the vertical displacement at peak load also increased when geogrid was placed in pervious concrete beam. In other words, the use of geogrid as reinforcement in pervious concrete delayed the occurrence of brittle fracture. Obviously, the improved flexural strength and increased vertical displacement at peak load led to the increase of energy absorption of the reinforced beam during pre-peak stage. However, the increment of vertical displacement at peak load of the geogrid-reinforced beam was lower than the increment of energy absorption during pre-peak stage (Table 4). As a result, the index $R_{e,p}$ of the geogrid-reinforced pervious concrete beam was above 1. This provides direct evidence that geogrid placed at an appropriate position in pervious concrete beam could delay the cracking and improve the stiffness at the pre-peak stage.

**Figure 15:**
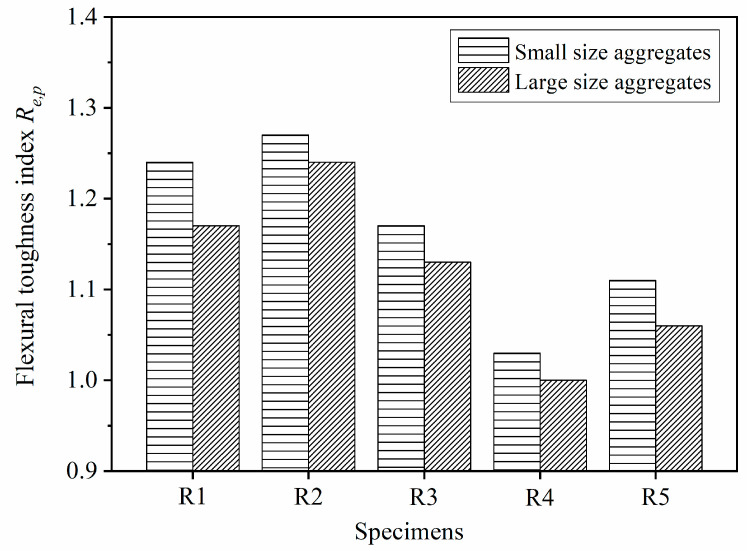
Toughness index $R_{e,p}$ of the geogrid-reinforced beam.

The influence of geogrid position and layer number on the pre-peak flexural performance of the geogrid-reinforced pervious concrete is also reflected by the pre-peak flexural toughness index $R_{e,p}$. Figure 15 shows that the magnitudes of pre-peak toughness index $R_{e,p}$ of the reinforced beams with different aggregate sizes were in the following order: R2 > R1 > R3 > R5 > R4. The geogrid-reinforced pervious concrete beam R2 had the highest $R_{e,p}$. The placement of geogrid at one-third the height of the pervious concrete beam could obtain the optimum flexural performance at the pre-peak stage. In addition, the index $R_{e,p}$ of the geogrid-reinforced pervious concrete beam decreased when the geogrid layer increased from one to two. The placement of geogrid decreased the conjoint points between aggregates in geogrid-reinforced concrete, resulting in an increase of porosity [22,33]. As mentioned in Section 3.1, the pervious concrete reinforced with two layers of geogrids had higher porosity than that reinforced with one layer of geogrid. While the gain of flexural strength provided by the second geogrids could not offset the loss of strength caused by the decreased conjoin points, the index $R_{e,p}$ of beams R4 and R5 were lower than beams R1 and R2, respectively. Furthermore, placing the geogrids at both the one-third and two-thirds heights could obtain better pre-peak flexural behavior when two layers of geogrids were placed in pervious concrete.

Figure 15 shows that the reinforced beams with small-size aggregates had higher $R_{e,p}$ than that with large-size aggregates. The geogrid used in this study had a mesh size of 40 mm × 40 mm. More 5–10 mm aggregates could fill in the apertures of geogrid as compared with the 10–15 mm aggregates. There were more conjoint points between the small-size aggregates in reinforced pervious concrete beam than that between large-size aggregates. As a result, the small-size aggregates were better interlocked with geogrids in pervious concrete beam thanks to the increased conjoint points as compared with the large-size aggregates. The good interlock between the geogrids and the small-size aggregates could make full use of the tensile strength of geogrid to restrain cracking and improve flexural strength, which resulted in the high index $R_{e,p}$ of the reinforced beam. It is apparent that the 5–10 mm aggregate is conducive to improving the pre-peak flexural behavior of the geogrid-reinforced pervious concrete beam as compared with the 10–15 mm aggregates.

### 5.3. Index $R_{e,m}$ of Geogrid-Reinforced Beam

**Figure 16:**
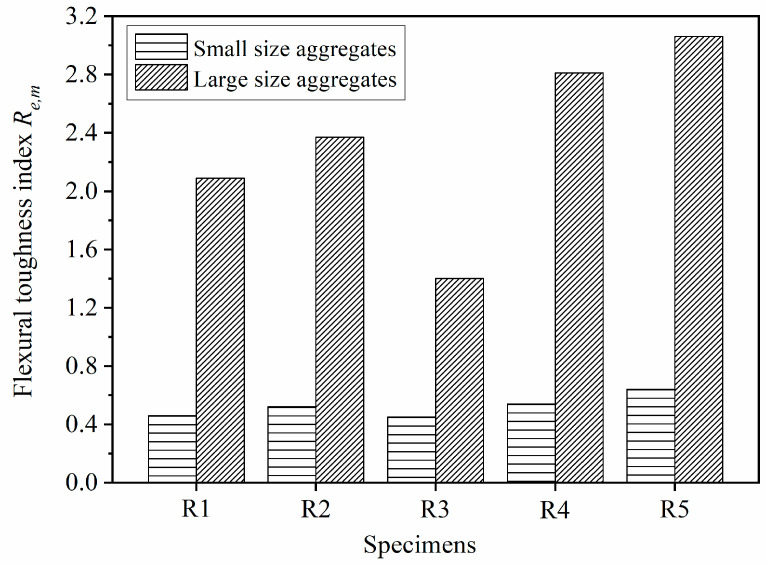
Toughness index $R_{e,m}$ of the geogrid-reinforced beam.

The flexural toughness index $R_{e,m}$ displays the flexural performance of the geogrid-reinforced pervious concrete beam at the post-peak stage during the bending test. Figure 16 illustrates the post-peak flexural toughness index $R_{e,m}$ of the geogrid-reinforced beam with different aggregate sizes. While the index $R_{e,m}$ of the reinforced beam with small-size aggregates was under 0.64, the index $R_{e,m}$ of the reinforced beam with large-size aggregates exceeded 1. The higher index $R_{e,m}$ indicates that the geogrid-reinforced beam with large-size aggregates had more remarkable post-cracking performance than that with small-size aggregates. Section 4 shows that different aggregates size led to different shapes of load-vertical displacement curves of pervious concrete beams. While the load of the reinforced beams with small-size aggregates at the post-peak stage was lower than the first peak load, the load of the beams with large-size aggregates in a large range of the post-peak stage was higher than the first peak load. The mesh size of the geogrid determined that a small amount of the large-size aggregates could be bonded and interlocked with the geogrid. The geogrid had little influence on the brittle failure of the pervious concrete beam due to the poor interlock between the geogrid and the large-size aggregates. After the cracking of pervious concrete beam with large-size aggregates, the geogrid played an important role in providing a crack-bridging effect, which resulted in the flexural toughness climbing to 376.7 J. Therefore, the geogrid-reinforced pervious concrete beam with large-size aggregates had higher index $R_{e,m}$ than the beam with small-size aggregates. However, the large-size aggregates led to lower pre-peak toughness index $R_{e,p}$ of geogrid-reinforced pervious concrete beam as compared with the small-size aggregates. Obviously, the effect of aggregate size on the pre-peak and post-peak flexural performance of the geogrid-reinforced pervious concrete beam is different. In summary, the large-size aggregates can provide noteworthy post-cracking performance for the geogrid-reinforced pervious concrete beam, while the small-size aggregates are helpful to improve the flexural strength.

There was an optimum position when geogrids were placed in pervious concrete beam to improve the flexural performance. Figure 16 shows that the magnitudes of the index $R_{e,m}$ of the beams with different aggregate sizes were in the following order: R5 > R4 > R2 > R1 > R3. Obviously, when one layer of geogrid was placed in pervious concrete beam, the geogrid placed at the one-third height was more suitable to improve the post-cracking performance than the geogrid placed at the one-fourth or middle height. The index $R_{e,m}$ of the reinforced beam SR2 and beam LR2 were 0.52 and 2.37, respectively. When the geogrid was placed at the middle height of the beam, the geogrid was far away from the tension zone. As a result, the geogrid could not bring its high tensile strength into full play to withstand the tension stress. The geogrid placed at a low geogrid position (e.g., one-fourth height) resulted in poor interlock between geogrid and pervious concrete. Therefore, the index $R_{e,m}$ of the geogrid-reinforced pervious concrete beams R1 and R3 are lower than the beam R2. When two layers of geogrids were placed in pervious concrete beam, the cracking resistance and flexural toughness of the pervious concrete beam could be improved due to the upper layer of geogrid. Different from the index $R_{e,p}$, the index $R_{e,m}$ of the beam reinforced with two layers of geogrids was higher than the beam reinforced with one layer of geogrid. Placing the geogrids at both the one-third and two-thirds heights of the pervious concrete beam could obtain the optimum post-crack performance.

## 6. Conclusions

This study focused on investigating the flexural performance and toughness characteristics of pervious concrete beam with different aggregate sizes, geogrid positions, and geogrid layer numbers. Four-point bending tests were conducted to evaluate the flexural strength, toughness, deformability, and energy absorption capability of the geogrid-reinforced pervious concrete beams. Based on the flexural toughness evaluation methods of the fiber-reinforced concrete, two dimensionless indices were proposed to evaluate the flexural toughness of the geogrid-reinforced pervious concrete. The following conclusions can be drawn:

1. The use of geogrid as reinforcement could improve the permeability of pervious concrete. Although the geogrid-reinforced pervious concrete had increased porosity (the permeability coefficient was above 4.5 mm/s), the geogrid could still enhance the compressive strength of pervious concrete.
2. The unreinforced pervious concrete beam was brittle and had no post-cracking performance. As compared with the unreinforced beams LU and SU, the flexural strengths of geogrid-reinforced pervious concrete beams with large- and small-size aggregates increased by 6% to 14% and 14% to 52%, respectively. Meanwhile, the geogrid-reinforced pervious concrete beams had remarkable post-cracking performance, with good deformability and energy absorption capability.
3. The interlocking effect between geogrids and aggregates is the main mechanism for the improved flexural performance of the geogrid-reinforced pervious concrete beam. The geogrid position and geogrid layer number are important factors to affect the flexural performance of pervious concrete. The optimum position for the geogrid was at one-third the height of the pervious concrete beam to improve flexural performance when one layer of geogrid was placed. The placement of the geogrids at both the one-third and two-thirds heights could obtain the optimum post-cracking performance of pervious concrete beam.
4. The coarse aggregate size could determine the interlocking effect between geogrids and aggregates to influence the flexural strength and load-vertical displacement curves shape of pervious concrete beam. While the small-size (5–10 mm) aggregates delayed the cracking and improved the flexural strength of geogrid-reinforced pervious concrete, the large-size (10–15 mm) aggregates played a significant role in obtaining noteworthy post-cracking performance.
5. The proposed flexural toughness indices distinguished the respective contributions of the geogrids and the aggregates to the flexural toughness of pervious concrete at the pre-peak and post-peak stages. Test results verify the excellent effectiveness and reliability of this proposed method. In addition, this method is not constrained by the first crack vertical displacement, loading condition, or specimen size. Therefore, it can be used to evaluate the flexural performance of pervious concrete with different geometrical dimensions and reinforcements.

## Figures and Tables

**Table 1:** Concrete mix proportions.

Material	Mass (kg/m^3^)
Coarse aggregate	1738
Cement	438
Fly ash	77
Water	134
Polycarboxylic superplasticizer	8

**Table 2:** Physical and mechanical properties of the biaxial geogrid.

Component	Description	Unit
Polymer type	polypropylene	None
Machine direction aperture size	40	mm
Transverse direction aperture size	40	mm
Mass per unit area	531	g/m^2^
Strength at 2% strain	10	kN/m
Strength at 5% strain	18	kN/m
Ultimate tensile strength	21	kN/m
Yield point elongation	5.5	%

**Table 3:** Sample variables.

Specimens	Geogrid Layer Number	Geogrid Position ^a^	Aggregate Size (mm)
SU	None	None	5–10
SR1	One	One-fourth	5–10
SR2	One	One-third	5–10
SR3	One	Middle	5–10
SR4	Two	One-fourth and three-fourths	5–10
SR5	Two	One-third and two-thirds	5–10
LU	None	None	10–15
LR1	One	One-fourth	10–15
LR2	One	One-third	10–15
LR3	One	Middle	10–15
LR4	Two	One-fourth and three-fourths	10–15
LR5	Two	One-third and two-thirds	10–15

^a^ Height of specimen.

**Table 4:** Energy absorption and toughness index.

Specimens	$\delta_{peak}\;(\mathrm{mm})$	$E_{peak}\;(J)$	$E_{post\cdot m}\;(J)$	$f_{c}\;(\mathrm{MPa})$	$f_{f}\;(\mathrm{MPa})$	$f_{p,r}\;(\mathrm{MPa})$	$f_{m,r}\;(\mathrm{MPa})$	$R_{e,p}$	$R_{e,m}$
SU	0.52	7.3		22.3	3.70	1.87			
SR1	0.60	10.4	54.5	26.3	4.21	2.33	0.87	1.24	0.46
SR2	0.52	9.3	61.6	27.4	5.12	2.39	0.97	1.27	0.52
SR3	0.56	9.2	52.9	23.2	5.61	2.19	0.84	1.17	0.45
SR4	0.55	7.9	63.7	24.1	4.34	1.92	1.01	1.03	0.54
SR5	0.53	8.2	76.2	29.3	5.23	2.07	1.20	1.11	0.64
LU	0.35	5.1		18.7	3.44	1.94			
LR1	0.37	6.3	261.5	25.5	3.76	2.27	4.04	1.17	2.09
LR2	0.38	6.8	296.3	26.3	3.92	2.40	4.58	1.24	2.37
LR3	0.34	5.6	176.4	19.9	3.83	2.20	2.72	1.13	1.40
LR4	0.50	7.3	347.5	21.8	3.60	1.95	5.45	1.00	2.81
LR5	0.54	8.3	376.7	25.0	3.65	2.05	5.94	1.06	3.06

Note: $f_{c}$ = unconfined compressive strength of the pervious concrete; $f_{f}$ = flexural strength of the pervious concrete beam.

## Data Availability

All data included in this study are available upon request by contact with the corresponding author.

## References

[1] Nguyen D.H., Sebaibi N., Boutouil M., Leleyter L., Baraud F. (2014). A modified method for the design of pervious concrete mix. Constr. Build. Mater..

[2] Yang J., Jiang G. (2003). Experimental study on properties of pervious concrete pavement materials. Cem. Concr. Res..

[3] Zhou J., Zheng M., Wang Q., Yang J., Lin T. (2016). Flexural fatigue behavior of polymer-modified pervious concrete with single sized aggregates. Constr. Build. Mater..

[4] Liu R., Chi Y., Jiang Q., Meng X., Wu K., Li S. (2021). Physical and mechanical properties of pervious concrete with multi-admixtures. Mag. Concr. Res..

[5] Rangelov M., Nassiri S., Haselbach L., Englund K. (2016). Using carbon fiber composites for reinforcing pervious concrete. Constr. Build. Mater..

[6] Kevern J.T., Biddle D., Cao Q. (2015). Effects of macrosynthetic fibers on pervious concrete properties. J. Mater. Civ. Eng..

[7] Hesami S., Ahmadi S., Nematzadeh M. (2014). Effects of rice husk ash and fiber on mechanical properties of pervious concrete pavement. Constr. Build. Mater..

[8] Rehder B., Banh K., Neithalath N. (2014). Fracture behavior of pervious concretes: The effects of pore structure and fibers. Eng. Fract. Mech..

[9] Akand L., Yang M., Wang X. (2018). Effectiveness of chemical treatment on polypropylene fibers as reinforcement in pervious concrete. Constr. Build. Mater..

[10] Chen C., Mcdowell G.R., Thom N.H. (2013). A study of geogrid-reinforced ballast using laboratory pull-out tests and discrete element modelling. Geomech. Geoeng..

[11] Chen R.P., Wang Y.W., Ye X.W., Bian X.C., Dong X.P. (2016). Tensile force of geogrids embedded in pile-supported reinforced embankment: A full-scale experimental study. Geotext. Geomembr..

[12] Lee K.M., Majunath V.R. (2000). Experimental and numerical studies of geosynthetic-reinforced sand slopes loaded with a footing. Can. Geotech. J..

[13] Karim M.R., Manivannan G., Gnanendran C.T., Lo S.-C.R. (2011). Predicting the long-term performance of a geogrid-reinforced embankment on soft soil using two-dimensional finite element analysis. Can. Geotech. J..

[14] Karpurapu R., Bathurst R.J. (1995). Behaviour of geosynthetic reinforced soil retaining walls using the finite element method. Comput. Geotech..

[15] Sun X., Han J., Kwon J., Parsons R.L., Wayne M.H. (2015). Radial stresses and resilient deformations of geogrid-stabilized unpaved roads under cyclic plate loading tests. Geotext. Geomembr..

[16] Khodaii A., Fallah S., Nejad F.M. (2009). Effects of geosynthetics on reduction of reflection cracking in asphalt overlays. Geotext. Geomembr..

[17] Hadi M.N., Al-Hedad A.S. (2020). Flexural fatigue behaviour of geogrid reinforced concrete pavements. Constr. Build. Mater..

[18] Meski F.E., Chehab G.D. (2014). Flexural behavior of concrete beams reinforced with different types of geogrids. J. Mater. Civ. Eng..

[19] Tang X., Higgins I., Jlilati M.N. (2018). Behavior of geogrid-reinforced Portland cement concrete under static flexural loading. Infrastructures.

[20] Al-Hedad A.S., Hadi M.N. (2019). Effect of geogrid reinforcement on the flexural behaviour of concrete pavements. Road Mater. Pavement Des..

[21] Mohamed R.N.A., El Sebai A.M., Gabr A.S.A.H. (2020). Flexural behavior of reinforced concrete slabs reinforced with innovative hybrid reinforcement of geogrids and steel bars. Buildings.

[22] Meng X., Chi Y., Jiang Q., Liu R., Wu K., Li S. (2019). Experimental investigation on the flexural behavior of pervious concrete beams reinforced with geogrids. Constr. Build. Mater..

[23] Deng Z., Shi F., Yin S., Tuladhar R. (2016). Characterisation of macro polyolefin fibre reinforcement in concrete through round determinate panel test. Constr. Build. Mater..

[24] Li J.J., Wan C.J., Niu J.G., Wu L.F., Wu Y.C. (2017). Investigation on flexural toughness evaluation method of steel fiber reinforced lightweight aggregate concrete. Constr. Build. Mater..

[25] Sukontasukkul P., Pongsopha P., Chindaprasirt P., Songpiriyakij S. (2018). Flexural performance and toughness of hybrid steel and polypropylene fibre reinforced geopolymer. Constr. Build. Mater..

[26] ASTM (1997). Standard Test Method for Flexural Strength Toughness and First Crack Strength of Fiber Reinforced Concrete.

[27] JSCE (1984). Method of Test for Flexural Strength and Flexural Toughness of Fibre Reinforced Concrete.

[28] Benthia N., Trottier J.F. (1995). Test methods for flexural toughness characterization of fiber reinforced concrete: Some concerns and proposition. ACI Mater. J..

[29] Jang S.J., Yun H.D. (2018). Combined effects of steel fiber and coarse aggregate size on the compressive and flexural toughness of high-strength concrete. Compos. Struct..

[30] Chen C., McDowell G.R., Thom N.H. (2012). Discrete element modelling of cyclic loads of geogrid-reinforced ballast under confined and unconfined conditions. Geotext. Geomembr..

[31] Gopalaratnam V.S., Gettu R. (1995). On the characterization of flexural toughness in fiber reinforced concretes. Cem. Concr. Compos..

[32] ASTM (2011). Standard Test Method for Flexural Performance of Fiber Reinforced Concrete (Using Beam with Third-Point Loading).

[33] Brown S.F., Thom N.H., Sanders P.J. (2001). A study of grid reinforced asphalt to combat reflection cracking. J. Assoc. Asph. Paving Technol..

